# The Blessed Doctrine of Rest

**Published:** 1883-07

**Authors:** 


### THE BLESSED DOCTRINE OF REST.

There are few practitioners, either general or special, who sufficiently recognize the therapeutical value of repose from labor. It is the very corner stone upon which rests the *vis medicatrix naturæ.* All nature has its alternate periods of activity and quiescence. Plants rest at night, many of the higher orders closing their petals or folding their leaves at nightfall only to open them again at the bidding of the morning sun. In addition to this they have their seasons of growth, and their winters of stillness. Even in tropical climates, where there are no chilling frosts to freeze the sap in their branches, deciduous plants drop their leaves and fall into slumber at stated periods of the year, to wake again with their energy renewed when they are sufficiently restored.

In man, as in all animals, the reparative process—the elimination of the effete and assimilation of the new—takes place mainly during rest. Deprive a man of sleep and he becomes weak and emaciated. Give him rest and he grows, and waxes strong. Infants require a great deal of sleep because growth is then most active. In mature life the greater the amount of exertion the longer should be the period of rest. The man who sleeps well transacts more business and does it better than he who at night vainly tosses upon his weary bed. Illness is in a large measure but the lack of necessary repose, either in an organ or of the whole system, and the successful physician is he who recognizes this fact and makes proper use of that knowledge. The hackneyed *vis medicatrix naturæ* is, after all, that upon which we must mainly rely for curing disease, and nature can only exert her healing powers during a condition of rest. In truth, she enforces this doctrine and enjoins repose in an exhausted or injured organ, by a soreness and tenderness which forbids use. The wounded animal holds up the hurt leg, and the lame horse refuses to move, or favors his weak limb by halting and limping in his gait. The broken arm is placed in a sling by the judicious surgeon, not alone that the bones may have an opportunity to knit by being kept in contact, but that the reparative process may be induced and hastened by the complete rest which is secured.

The value of a prolonged course of athletics has been seriously questioned by some authors, because it was stated that while such exercise strengthened certain organs, it weakened others. It was noticed that in the English universities, many of the students who were members of the boating and cricket clubs were the victims of a sudden death. Numbers of the most stalwart and robust of the graduates, those who had, it was supposed, cultivated their bodies to the very highest point of physical perfection, died soon after graduation, of heart disease. So persistently had they trained and exercised themselves during the latter part of their undergraduate term, in anticipation of the matches which occurred simultaneously with their last commencement, that the heart had been overworked, had not been given sufficient opportunity for the partial rest which was necessary to its perfect function, had become hypertrophied, had weakened, and finally failed because of a lack of sufficient repose for recuperation. The observation of these truths has resulted in an entire change of the method of training for athletic contests.

To the dentist, a consideration of these facts is of importance, because there is many an ill which only needs the application of this doctrine of rest to afford entire relief. We have known those who were great sufferers from oral diseases because of their continual chewing of gum. Many of the dental ills of persistent tobacco chewers arise from their almost continual use of the organs of mastication. Some of the very worst cases of exostosis, of irritation and calcification of the dental pulp, of hypertrophies and absorptions which we ever met, were in the mouths of this latter class. Except in the later stages of dental irritation, the teeth of tobacco chewers are usually very firmly set in their sockets, the result of hypertrophies induced by over exercising the jaws and teeth. Later on, the tissues gave way under the inordinate labor, and absorption of the alveolus with denudation loosening and final falling out of the teeth succeeds. When this last stage threatens, it may frequently be aborted by leaving off the use of tobacco and giving the jaws comparative rest.

When a part of the teeth are lost, extra labor is usually thrown upon those which remain, and dental irritation inevitably follows sooner or later.

It is not very long since a gentleman consulted us upon a soreness with persistent gnawing pains in the teeth and their investing tissues of one side of his face. An examination showed that the only ones which occluded in such manner as to afford him a good masticating surface were the second and third molars of the left side. He was fond of hard, solid food, and mainly subsisted upon it, and these teeth showed indication of the hard usage to which they had been put. A partial set of artificial teeth was made for him, which antagonized with others of the natural ones in such a manner as to prevent the occlusion of the lame teeth, by not allowing the jaws to be sufficiently closed for that purpose. In spite of the annoyance of the plate he persisted in its use for two months, when all signs of pain and irritation had departed, and he was advised to leave it out. Certain other teeth were filled and their lost contour so restored as to partially relieve the formerly overworked organs, since which time he has had no return of the annoyance.

Another case was that of a man upon whose bicuspids on one side the whole burthen of labor was thrown. He was advised to change his diet to one less difficult of mastication, that partial rest might be secured, and this was followed by almost immediate relief.

Severe periodontitis has been relieved by moulding warm gutta percha about some of the teeth on each side the affected one. When cool this was removed and trimmed to proper shape, and then replaced to act as a block to prevent the occlusion of the sore one until it had a chance to recover.

Instances of the application of this doctrine of rest in dental practice might be multiplied indefinitely, but the intelligent reader who has been patient enough to follow this far will scarcely need them. He will understand to what cases the law is applicable, and not confound them with those in which teeth are being lost through absolute lack of exercise.

